# A multivariate analysis of canonical and non-canonical uses of switch-reference markers in Mbyá narratives

**DOI:** 10.1515/cllt-2024-0015

**Published:** 2024-12-05

**Authors:** Guillaume Thomas, Germino Duarte

**Affiliations:** Department of Linguistics, 7938 University of Toronto, Toronto, Canada; Independent, 25 de Mayo, Argentina

**Keywords:** switch-reference, Mbyá Guaraní, penalized regression

## Abstract

Switch-reference is a family of grammatical devices whose primary function is to indicate whether two linked clauses have coreferential pivots, where the pivot is a prominent argument in each clause. In some languages, in addition to their function of reference tracking, switch-reference markers can be used to indicate whether the events or situations described by the two linked clauses differ with respect to some parameter, such as time, place or actuality. This phenomenon is known as non-canonical switch-reference. Whether canonical and non-canonical switch-reference marking are distinct grammatical phenomena is still an open question. In this paper, we investigate uses of switch-reference markers in a corpus of Mbyá Guaraní (Tupian) narratives, and we argue that the alternation between canonical and non-canonical uses is an epiphenomenon of the multifactorial and probabilistic nature of switch-reference marker choice. In this perspective, there is only one grammatical process of switch-reference marking and the distinction between canonical and non-canonical switch-reference marking is matter of language use.

## 1 Introduction

This paper discusses the relation between canonical and non-canonical uses of switch-reference markers based on a case study of Mbyá Guaraní. Switch-reference (henceforth: SR) has been defined as “a morpheme associated with clause junctures that indicates whether a prominent argument in each clause co-refers” (McKenzie 2014; cf. Haiman and Munro 1983). We refer to the clause that contains the SR marker as the *marked clause*, and to the other one as the *reference clause*. In the following examples from Amele, the same (SM) suffix *-me* in (a) indicates coreference between the subject of the reference clause *na* ∅*-i-te-i-a* (‘he gave me the stick’) and the subject of the marked clause *uqa q-it-i-me-i* (‘he hit me’). The different (DF) suffix *-co* in (b) indicates disjoint reference between the subjects of the marked and reference clauses. Following Stirling (1993), we use the term *pivots* to refer to the two arguments that are related by SR marking.

(1)

**Table:** 

**Amele** (Stirling 1993: 184)

a.

**Table:** 

*Uqa*	*q-it-i-me-i*	*na*	∅*-i-te-i-a.*
3sg	hit-lsg-pred-sm-3sg	stick	give-pred-1sg-3sg-todP
‘He hit me and then gave me the stick.’

b.

**Table:** 

*Hina*	*ho-co-m*	*sab*	*je-i-a.*
2sg	come-df-2sg	food	eat-3sg-todP
‘You came and he ate the food.’

It is well known that in some languages, SR markers can be used to indicate whether the situations described by two clauses are similar or not, independently of coreference between pivots. This phenomenon is known as non-canonical switch-reference and is illustrated in (2), where a different marker is used despite pivot coreference, in order to indicate a change of place:

(2)

**Table:** 

**Amele** (Stirling 1993: 216)						
*Age*	*ceta*	*gul-do-co-bil*	*1-i*	*bahim*	*na*	*tac-ein*
3pl	yam	carry-3sg-df-3pl	go-pred	floor	on	fill-3pl.RemP
‘They carried the yams on their shoulders and went and filled up the yam store.’						

Given the existence of non-canonical uses of SR marking, some scholars have argued that the function of SR constructions is to mark thematic continuity or discontinuity across clauses, referential continuity being only one aspect thereof (see a.o. Mithun 1993, 2021; Pustet 2013; Stirling 1993; van Gijn 2012, 2016a, 2016b; Watkins 1993). From this perspective, canonical and non-canonical SR marking are understood as different uses of the same construction, which emphasize referential or non-referential dimensions of thematic continuity. Here, we adopt Givón’s (2001) definition of thematic coherence or continuity:

(3)

**Table:** 

**Coherence as continuity** (Givón 2001: 328–329):
Coherence is the continuity or recurrence of some element(s) across a contiguous span of multi-propositional discourse.

Givón proceeds to list seven elements that can contribute to thematic continuity: referents (‘participants’), location, temporality, aspectuality, modality, perspective (‘narrative voice’) and action/events.1Following Givón, we understand referential continuity to be restricted to reference to participants of events described in discourse. I.e., continuous reference to non-participants such as times does not fall under the label ‘referential continuity.’ This is merely a terminological point and not a denial that one may refer to times, events and such. Under the view of SR constructions as indicators of thematic continuity, canonical uses of SR markers would attend to the first of these elements, while non-canonical uses would attend to others.

This literature raises the question of how to model the process of SR marker choice in such a way as to account for both canonical and non-canonical uses. The present manuscript addresses this question through a case study of SR marking in a corpus of narratives produced in Mbyá Guaraní, a Tupian language spoken by about 30,000 speakers in Argentina, Brazil and Paraguay (Ladeira 2018), in which canonical uses of SR markers track subject coreference (Dooley 1989, 1992, 1999).

In the first part of the manuscript, we explore the distribution of canonical and non-canonical uses of SR markers in our corpus. We evaluate the contribution of various factors to predicting the use of same versus different markers using monofactorial tests and we motivate an analysis of SR marker choice in Mbyá as a probabilistic process that encompasses both canonical and non-canonical uses, eschewing the reduction of non-canonical uses to non-referential dimensions of thematic continuity. In the second part of the manuscript, we show that a multifactorial model of SR marker choice that is blind to the distinction between canonical and non-canonical uses will still predict non-canonical uses of SR markers with adequate frequency and in adequate contexts. We conclude from these results that the distinction between canonical and non-canonical uses of SR markers in Mbyá can be adequately modelled as a side effect of a single multifactorial process of SR marker choice.

The paper is structured as follows. In Section 2, we give an overview of previous studies of SR in Mbyá. In Section 3, we introduce our corpus and the variables to be used for the analysis of SR. In Section 4, we explore the corpus and motivate a probabilistic analysis of SR marker choice. In Section 5, we fit a model of SR marker choice to the corpus. We argue that this model provides an adequate analysis of both canonical and non-canonical uses of SR markers. Section 6 concludes.

## 2 Overview of switch-reference in Mbyá Guaraní

The SR system of Mbyá was studied notably by Dooley (1989, 1992, 1999). Switch-reference is expressed by the particles *vy* (SS) and *ramo* or its reduced form *rã* (DS), as illustrated in examples (4) and (5).2A list of glosses is provided in appendix. Note that Mbyá Guaraní agreement follows an active-inactive alignment pattern. Intransitive verbs agree in person and number with their subject, different agreement markers being used for active verbs and inactive verbs. Transitive verbs agree either with their highest argument on the person hierarchy 1 > 2 > 3, or with their subject in case of a tie. Agreement with transitive subjects is expressed with active agreement markers, and agreement with objects is expressed with inactive agreement markers. In glosses, ‘A3’ stands for ‘third person agreement marker, active class’ and ‘B3’ stands for ‘third person agreement marker, inactive class.’

(4)

**Table:** 

*Ava*	*o-o*	***vy***	*mboi*	*o-exa.*	
man	a3-go	sm	snake	a3-see	
‘When the man_*i*_ went, he_*i*/*j_ saw the snake.’					(Dooley 1989)

(5)

**Table:** 

*Ava*	*o-o*	***ramo***	*mboi*	*o-exa.*	
man	a3-go	df	snake	a3-see	
‘When the man went, the snake saw him.’					(Dooley 1989)

In such constructions, SR markers track the reference of subjects (Dooley 1989). Dooley (2015: 25) identifies the subject with the nominative argument a verb, i.e., the unique argument of intransitive verbs (S) or the (proto-)agent argument of (di)transitive verbs (A), and he discusses three constructions that are sensitive to this grammatical function, in addition to SR marking: (i) reflexive possessive markers must be bound by subjects, (ii) impersonal voice eliminates reference to the subject argument and (iii) the subject of post-verbal converbs must be coreferent with the subject of the verb they modify.

Dooley (1989) argues that SR in Mbyá tracks the reference of grammatical subjects rather than agents or topics. In example (6), the same marker *vy* indicates that the grammatical subjects of *okaru* (‘eating’) and *tove tomano* (‘let him die’) corefer, although the subject of *okaru*, which is also its agent, does not corefer with the addressee, which is the notional agent of the optative predication *tove tomano*:3Note that in this example, unlike in previous ones, the marked clause follows the reference clause. The preferred position of the marked clause varies with the semantic relation between marked and reference clauses, see Dooley (2016: §21.3.1).

(6)

**Table:** 

*Pe-juka*	*e’ỹ*	*teĩ*	*tove*	*t-o-mano*	*ha’e*	*ae*	*o-karu*	*e’ỹ*	*vy.*
a2pl-kill	neg	conc	opt	opt-a3-die	3	int	a3-eat	neg	sm
‘Without your killing him, let him die all by himself from not eating.’									
(Dooley 1989: 7)									

In example (7), the different marker *ramo* indicates that the grammatical subjects of *omombe’u* (‘he talked’) and *aexa* (‘I saw’) do not corefer, although both clauses share the same topic, *compadre Galdino*, which is the object of the marked clause and the subject of the reference clause:

(7)

**Table:** 

*Compadre*	*Galdino*	*ma*	*a-exa*	*Roberto*	*r-o*	*py*	*ramo*	*ma,*	*gu-a’y-’i*
godfather	Galdino	bdy	a1sg-see	Roberto	r-house	in	df	bdy	refl-son-dim

*o-mombe’u.*									
a3-talk									
‘Compadre Galdino, when I saw him at Roberto’s house, he talked about his little son.’ (Dooley 1989: 10)									

With respect to their external syntax, Dooley (2015: 119) argues that marked clauses in SR constructions are adverbial subordinate clauses. Note that SR markers underspecify the semantic relation between the marked and reference clauses. To illustrate, while the marked clauses in examples (4) and (5) are interpreted as temporal adverbial clauses, causal or conditional interpretations of SR constructions are also attested, among other interpretations.

The SR constructions we discussed up to this point relate a marked clause to a reference clause by subordination. SR markers are also attested sentence initially with the anaphoric pronoun *ha’e* in place of a marked clause, as illustrated by examples (8) and (9), where *ha’e* is anaphoric to the previous sentence. We call these construction *reduced switch-reference*. By contrast, we refer to SR constructions like those illustrated by (4) and (5) as *full switch-reference*.

(8)

**Table:** 

*Peteĩ-gue*	*je*	*ava*	*o-o*	*o-i-ny*	*t-ape*	*r-upi.*
one-time	hsy	man	a3-go	a3-be-conv	npossd-road	r-loc
***Ha’e***	***vy***	*je*	*o-exa*	*apere’a.*		
ana	sm	hsy	a3-see	preá		
‘Once, a man was going on a road. He saw a preá.’						(Veríssimo 2002a)

(9)

**Table:** 

*Guaxu*	*je*	*o-po-opo*		*o-iko-vy,*		*nd-o-guapy-i,*		*nd-o-pyta-i*		*guive.*
deer	hsy	a3-jump-red		a3-be-conv		neg-a3-sit-neg,		neg-a3-stop-neg		add

***Ha’e***	***rã***	*je*	*irũ*	*kuery*	*o-porandu:*	*“Mba’e*	*tu*	*r-endu”*	*he’i.*
ana	df	hsy	friend	pl	a3-ask	what	mir	a2sg-feel	a3.say
‘A deer was jumping around, it couldn’t sit, it couldn’t stand still either. So his friends asked – What is wrong with you?’ (Veríssimo 2002a)									

Note that in this pair of examples, the SR markers *vy* and *rã* occur at the beginning of a new sentence, and they indicate whether the subject of that sentence corefers with the subject of the previous sentence. In example (8), the subjects of both sentences refer to the man introduced in the first sentence, and a same marker is used. In example (9), the subject of the first sentence introduces a deer, and the subject of the second sentence refers to his friends. A different marker is used.

Reduced SR is part of a broader class of sentence initial connectives formed by combining the anaphoric pronoun *ha’e* with a subordinating conjunction or postposition, as illustrated by the connective *ha’e gui* in example (10):

(10)

**Table:** 

*Peteĩ*	*ára*	*je*	*Vera*	*o-o*	*yakã*	*py*	*pira*	*o-jopoi*	*vy.*
One	day	hsy	Vera	a3-go	river	to	fish	a3-feed	sm
***Ha’e***	***gui***	*je*	*pira*	*o-gueno-ẽ*			*ma.*		
ana	src	hsy	fish	a3-comit-leave			asp		
‘One day, Vera went to the river to fish, and he caught a fish.’ (Veríssimo 2002a)									

Dooley (1992, 2015) calls these constructions *reduced subordinate clauses* and argues that the pronoun *ha’e* refers to the content of a preceding discourse unit. In example (10), *ha’e* is anaphoric to the first sentence, and the postposition *gui* indicates that the event described in the second sentence (Vera caught a fish) is contingent on the event described in the first one (Vera went to the river). In other words, reduced subordinate clauses appear to be a form of tail-head linkage, a phenomenon that has been documented in multiple language families and areas (Guérin 2019) and that has been independently observed to interact with SR (Guillaume 2011).

In all previous examples, SR markers are used canonically: the choice of marker tracks subject reference. With reduced SR markers in examples (6) and (7), the subjects whose reference is being tracked are those of the reference clause and of the preceding sentence, which serves as the antecedent of the anaphoric pronoun *ha’e*. Non-canonical uses of SR markers are also attested in both types of SR constructions, as illustrated by examples (11) and (12). For ease of reading, we gloss non-canonical uses of **vy** and **rã**∼**ramo** as sm_nc_ and df_nc,_ respectively:

(11)

**Table:** 

*Nda-xe-ayvu*	*kuaa-i*	*r-e*	***ramo*** *,*	*a-iko*	*tema.*
neg-b1.sg-speak	know-neg	R-abl	df_nc_	a1.sg-live	cont
‘Even though I didn’t know how to speak, I got along’ (Dooley 2011).

(12)

**Table:** 

*Ha’e*	*kuery*	*ma*	*je*	*o-ma’ẽ*	*guaxu*	*’rã*	*o-ke*	*vy.*	
ana	pl	bdy	hsy	a3-look	big	hab	3-sleep	sm	
***Ha’e***	***rã***	*nd-o-ke-i*		*vy*	*ma*	*je*	*o-ma’ẽ*		*r-a’y-’i.*
ana	df_nc_	neg-a3-sleep-neg		sm	bdy	hsy	a3-look		r-small-dim
‘When they [owls] sleep, their eyes are wide open. But when they are awake, their eyes are narrow.’ (Veríssimo 2002b)									

In example (11), the different marker *ramo* is used despite the coreference of the subjects of the marked and reference clauses. In example (12), *ha’e rã* relates two sentences with coreferential subjects. Non-canonical uses of SR markers are also attested with the same marker *vy*.

## 3 Corpus and variables

Our corpus consists of 81 narratives from the state of Parana and São Paulo in Brazil. It includes 1,313 sentences (14,575 tokens). The narratives were produced by seven adult Mbyá speakers from the state of Paraná. A first part of the corpus consists of 33 narratives composed in various workshops organized by Robert Dooley and the Summer Institute of Linguistics between 1976 and 1990. An interlinearized version of the corpus with a translation into English is available on the Archive of the Indigenous Languages of the America (Dooley 2011). The second part consists of 48 narratives collected in Veríssimo (2002a, 2002b) together with their translation into Brazilian Portuguese. Narratives in both part of the corpus have been used as educational material. This is especially true of narratives in the second part, which were created for literacy training. This may explain the short length of most narratives in the corpus, which averages 16 sentences per narrative.

Several layers of annotations were added by the first author and collaborators: interlinear glosses when missing, syntactic structure in dependency grammar, coreference relation and animacy (see Thomas et al. 2021). For this study, we added a layer of rhetorical relation annotations between discourse units that are related by a switch-reference marker. Rhetorical relation annotation was carried out collaboratively by the two authors, using consensus judgment. In doing so, we relied on the second author’s interpretation of the narratives as a native speaker of Mbyá as well as annotation guidelines described in Section 3.2. We extracted 762 occurrences of SR constructions from the corpus: 392 full SR constructions, 370 reduced SR constructions. Of these, 368 use same markers and 394 use different markers. 16 occurrences of SR markers are used non-canonically. We say that a different marker is used non-canonically when the subjects of the SR construction corefer, and a same marker is used non-canonically when they don’t.

Each observation in our study corresponds to a single occurrence of an SR construction. Relevant properties of these observations are coded as values of a series of variables. A first variable, marker_type, encodes the form of the SR marker in an observation. It has two levels: same (if the SR marker is *vy*) or different (if the SR marker in the observation is *ramo* or *rã*). Note that these two levels only indicate the form of the SR marker (*vy* versus *ramo* or *rã*). Whether the pivots in a given SR construction are coreferential or not is coded by a different set of variables described in Section 3.1.

We define 7 variables that are potential predictors of SR marker choice. These variables can be grouped into 5 classes: referential continuity, rhetorical relations, mirativity, spatio-temporal continuity and construction type. We discuss these five classes of variables in the following subsections.

### 3.1 Referential continuity

We hypothesize that SR marking in Mbyá may be sensitive to referential continuity beyond pivot coreference. We define a variable referential_continuity, which captures a subset of coreference relations between the marked clause and the reference clause of an SR construction. However, since the marked clause can either precede or follow the reference clause, it is more convenient to define this variable using the concepts of *anteceding discourse unit* and *current discourse unit*:

(13)

**Table:** 

Anteceding and current discourse units:
Given a switch-reference marker that relates two discourse units, the current discourse unit is the one that was uttered last, and the anteceding discourse unit is the one that was uttered first.

Discourse units (DUs) are defined in segmented discourse representation theory, the theory of discourse structure that underlies our treatment of rhetorical relations (see subsection 3.2). With full SR, the discourse units related by SR markers are the marked clause and the reference clause. With reduced SR, the discourse units related by SR are the reference clause and the propositional antecedent of the anaphoric pronoun *ha’e*.

In order to measure referential continuity beyond subject coreference, we borrow the notion of backward looking centre from centering theory (Grosz and Sidner 1986; Grosz et al. 1995), redefining it for the purposes of the present study. In the following definition, it is assumed that referential expressions are ranked by prominence in a clause according to their grammatical function, where subjects are more prominent than objects, which in turn are more prominent than obliques and adjuncts:

(14)

**Table:** 

Backward Looking Centre:
The backward looking centre (BC) of a switch-reference construction is the referential expression in the current DU that has the most prominent antecedent in the anteceding DU.

With these definitions in place, we can define the variable referential_continuity, which has 4 levels ordered by decreasing continuity (SS > SO.OS > OO > none):

(15)

**Table:** 

referential_continuity

a.

**Table:** 

SS: the BC is the subject of the current DU, and its antecedent is the subject of the anteceding DU.

b.

**Table:** 

SO.OS:
*either* the BC is the subject of the current DU, but its antecedent is not the subject of the anteceding DU,
*or* the BC is not the subject of the current DU, but its antecedent is the subject of the anteceding DU.

c.

**Table:** 

OO: the BC is not the subject of the current DU, and its antecedent is not the subject of the anteceding DU.

d.

**Table:** 

none: no referential expression in the current DU has an antecedent in the anteceding DU.

Borrowing again from centering theory, we hypothesize that coreference between more prominent arguments generates more discourse coherence, which is captured in the ordering of the levels of this variable. The highest degree of referential continuity, SS, corresponds to coreference between the canonical pivots of the SR construction. We expect that higher levels of referential continuity will be associated with a higher proportion of same marker use.

Example (16) illustrates our use of referential_continuity. The SR marker *ramo* relates the anteceding DU labelled *π*_*a*_ to the current DU labelled *π*_*b*_. The BC of this SR construction is the dative argument of *π*_*b*_ (*xevy*, ‘to me’), and its antecedent is the subject of *π*_*a*_. Its reference is the narrator. The subject of *π*_*b*_ (*mamaẽ*, ‘mother’) is not the BC, since its antecedent in *π*_*a*_ is less prominent than the antecedent of *xevy*. The level of referential_continuity for the SR construction (*π*_*a*_, *π*_*b*_) is SO.OS:

(16)

**Table:** 

*Ha’e*	*rire*	*ma*	*xee*	*ma*	*a-ju*	*vy*	${[}_{\pi_{a}}$	*mamaẽ*	*pe*	*a-porandu*	.]	*Ha’e*_*a*_	***ramo***
ana	seq	bdy	b1.sg	bdy	a1.sg-come	sm		mother	dat	a1.sg-ask		ana	df

*ma*	${[}_{\pi_{b}}$	*mamaẽ*	*aipo-e’i*	*xe-vy*	]	:	“	*O-o*	*guyra-’i*	*avy*	*vy*	*”*	*he’i*
bdy		mother	attn-say	b1.sg-dat				a3-go	bird-dim	hunt	sm		a3.say
‘After a while, I went and asked my mother. She told me: “He went bird hunting”, she said.’ (Dooley 2011)													

### 3.2 Rhetorical relations

Several prominent approaches to discourse interpretation argue that a discourse is perceived as coherent only if its utterances are connected by rhetorical relations (also known as coherence relations) in a tree-like or graph-like discourse structure (Asher and Lascarides 2003; Hobbs 1985; Mann and Thompson 1988). In his study of reduced SR in Mbyá, Dooley (1989) noted that the use of different SR markers appears to be favoured by relations of contrast and counter-expectation between the discourse units related by SR markers. This was illustrated with examples (11) and (12) in Section 2. In the present study, we hypothesize more generally that SR markers relate discourse units that are connected by a rhetorical relation, and that the nature of this relation may affect the speaker’s choice of SR marker.

We analyze rhetorical relations using segmented discourse representation theory (SDRT; Asher and Lascarides 2003). SDRT is built on top of a dynamic semantics (discourse representation theory, see Kamp and Reyle 1993), to which it adds speech act discourse referents and rhetorical relations between them. Speech act discourse referents (SA-drefs) label the content of clauses and other discourse units (DUs).

**Figure 1:**
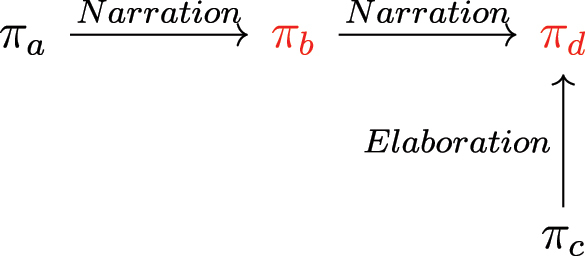
Simplified discourse structure of example (17).

Since they have propositional content, the marked and reference clauses related by SR markers are DUs that introduce SA-drefs. The same goes for the antecedent of the anaphoric pronoun *ha’e* in reduced SR. We can therefore situate the DUs related by SR markers on an SDRT graph. To illustrate, consider discourse (17) and its simplified discourse structure in Figure 1. The first occurrence of the same marker *vy* relates the marked clause *eixu rugue oẽmba* (‘the wasps got out’) to the reference clause *xepipa rive* (‘they stung me’). These clauses introduce the SA-drefs *π*_*a*_ and *π*_*b*_, respectively, which are related by *Narration*. The second occurrence of *vy* relates the pronoun *ha’e* to its reference clause. *Ha’e* is anaphoric to *xepipa rive*, and the reference clause is the matrix clause of the second sentence, *xero katy ajevy* (‘I came back home’), which introduces the SA-dref *π*_*d*_. This SA-dref relates to *π*_*b*_ by *Narration*. The SA-dref *π*_*c*_ is introduced by the clause *aja’eo reve* (‘crying’), which is adjoined to the reference clause of the SR construction. We relate *π*_*c*_ to *π*_*d*_ with a relation of *Elaboration*, *π*_*c*_ being subordinate to *π*_*d*_ in the discourse structure.4Note that *π*_*c*_ could also be analyzed as relating to *π*_*d*_ by the relation *accompanying circumstance*, which has been proposed for the analysis of participial adjuncts in English. See Behrens and Fabricius-Hansen (2010) and Behrens et al. (2012) for a discussion of this relation and the analysis of participial adjuncts in SDRT.


(17)

**Table:** 

*Ha’e*	*ramo-ve*	${[}_{\pi_{a}}$	*eixu*	*r-ugue*	*o-ẽ-mba*	***vy***	]	${[}_{\pi_{b}}$	*xe-pi-pa*	*rive*	]
ana	ds-int		wasp	r-swarm	a3-get.out	ss			b1.sg-sting-comp	excl	

*Ha’e*_*b*_	***vy***	${[}_{\pi_{c}}$	*a-jae’o*	*r-eve*	]	${[}_{\pi_{d}}$	*xe-r-o*	*katy*	*a-jevy*	] …	
ana	ss		a1.sg-cry	r-man			b1.sg-r-house	dir	a1.sg-come.back		
‘The wasps got out and stung me all over. I came back home crying (…)’ (Veríssimo 2002b)											

Every occurrence of an SR construction in the corpus was annotated for its rhetorical structure by the two authors. This annotation was based on consensus judgments between the second author, who provided expertise in the interpretation of narratives as a native speaker of Mbyá, and the first author, who provided expertise in theories of discourse semantics. The annotation followed the guidelines for SDRT annotations of Reese et al. (2007). Our annotation was constrained by the requirement that any two discourse units connected by an SR marker should be related by a unique rhetorical relation. In cases where several rhetorical relations were considered to be plausible candidates, the relation that was judged to be more salient by the second author was chosen.

Because of the large number of rhetorical relations in our inventory (see Table 1 below), some rhetorical relations are quite rare in the corpus. In order to mitigate this scarcity, rhetorical relations were grouped into coarser classes along the dimensions of additivity and polarity introduced by Sanders et al. (2021).5Sanders et al. present a classification of rhetorical or coherence relations that is applicable to annotation schemas used in major theories of discourse structure, including SDRT. The dimensions used in their classification are *polarity*, *basic operation*, *source of coherence*, *implication order* and *temporality*. Out of these five dimensions, we determined that only *polarity*, *basic operation* and *temporality* are relevant to our study of SR marking. In this process, we were guided by the hypothesis that the function of SR marking is to mark thematic continuity across discourse units. *Source of coherence* distinguishes between rhetorical relations that connect discourse units at the level of their propositional content, and rhetorical relations that express the speaker’s opinion, argument, claim or conclusion. This dimension appears to be orthogonal to the distinction between thematic continuity and discontinuity and therefore irrelevant to the present study. *Implication order* only applies to a subset of rhetorical relations, which raises issues for the statistical modelling of SR marker choice. In addition, it also appears to be orthogonal to thematic (dis)continuity, since it merely distinguishes causal relations that introduce the cause first from those that introduce the consequence first. We attend to *temporality* as part of a broader treatment of spatio-temporal continuity, see Section 3.3. This leaves us with two dimensions, *polarity* and *basic operation*, the second of which we rename as *additivity* for conciseness and clarity.


**Table 1:** Classification of rhetorical relations by additivity and polarity.

additivity			polarity		
additive	causal	additive/causal	positive	negative	positive/negative
Attribution	Consequence	Alternation	Attribution	Contrast	Alternation
Background	Explanation	Contrast	Background		
Commentary	Goal	Elaboration	Commentary		
Continuation	Result		Consequence		
Narration			Continuation		
Precondition			Elaboration		
Source			Explanation		
			Goal		
			Parallel		
			Precondition		
			Result		
			Source		

The variable polarity has two levels, positive and negative. Negative rhetorical relations include contrastive, adversative and concession relations. In our inventory of rhetorical relations, only *Contrast* is unambiguously negative. Sanders et al. (2021) also discuss a negative use of the *Alternation* relation in SDRT, which is notably expressed by exclusive disjunction in English. All other relations are positive. The following examples illustrate negative polarity relations with a canonical and a non-canonical use of SR markers, respectively:

(18)

**Table:** 

${[}_{\pi_{a}}$	*A-j-exa-uka*	*ta*	*ra’aga*	] ${[}_{\pi_{a}}$	***vy***	*rive*	*ta’vy*	${[}_{\pi_{b}}$	*xee*	*ae*	*a-je-juka*
	a1.sg-ref-see-caus	prosp	cf		sm	excl	frust		I	int	a1.sg-refl-kill

*rai*	.]_*b*_								
almost									
‘I was just going to show off, but I almost got myself killed.’ (Veríssimo 2002a)

(19)

**Table:** 

${[}_{\pi_{a}}$	*Nda-xe-ayvu*	*kuaa-i*	*r-e*	]	***ramo***	${[}_{\pi_{b}}$	*a-iko*	*tema*	].
	neg-b1.sg-speak	know.how-neg	r-abl		df_nc_		a1.sg-live	cont	
‘I didn’t know how to speak, but I survived.’ (Dooley 2011)									

Positive rhetorical relations have already been illustrated in previous examples, see for instance *Narration* and *Elaboration* in example (17). Because negative relations express a form of thematic rupture (such as the frustration of an expected outcome), we expect that, everything else being equal, discourse units related by a negative relation will have a higher proportion of different markers than discourse units related by a positive relation.

The variable additivity has two levels, additive and causal. Rhetorical relations that are causal all involve an implicational relation, while additive ones do not. The SDRT relations *Result*, *Explanation* and *Consequence* are causal, and the relations *Alternation*, *Contrast* and *Elaboration* have causal and additive uses. All other relations are additive. Example (20) illustrates the use of causal relations, in this case *Explanation*. We expect that, everything else being equal, discourse units related by a causal relation will have a higher proportion of different markers than discourse units related by a non-causal relation.

(20)

**Table:** 

${[}_{\pi_{a}}$	*Urutau*	*ma*	*je*	*nd-o-vy’a-i*	]	${[}_{\pi_{b}}$	*i-juru*	*guaxu*	*vaipa*	]	***vy***	.
	potoo	bdy	hsy	neg-a3-happy-neg			b3-mouth	big	very		sm	
‘The potoo was unhappy because he had such a large mouth.’ (Dooley 2011)												

Table 1 lists the rhetorical relations used for annotation, grouped by levels of additivity and polarity.6Note that the rhetorical relation *goal* is absent from Reese et al. (2007) and was adopted from Muller et al. (2012).


### 3.3 Spatio-temporal continuity

In some SR systems, different markers can indicate spatial or temporal discontinuity (see Roberts 2017; Stirling 1993). Roberts (1988) notably showed that in Amele, different marking can be used to indicate a change of place or time, among other parameters. Note that in that case, the factor that conditions the use of sm versus df marking is the spatio-temporal identity of the situations described in the discourse units related by SR. Specifically, different marking can be used to indicate that two situations unfold at different times or in different places. We capture this form of spatio-temporal continuity through two variables, time and place.

The variable time has two levels: sequence and containment. The former is used when the situations described by the marked and reference clauses unfold at different times, that is to say, they follow one another in time (regardless of the lapse between them). The latter was used when the two situations are simultaneous, or when one situation includes the other in time.7If an interval B extends past an interval A in time, interval A and B are said to stand in a sequence relation, even if the two intervals have a non-empty intersection. In other words, sequence stands for partial rather than complete precedence in a period structure. Note that several rhetorical relations in SDRT have temporal entailments. The most frequent ones in our corpus are *narration*, which entails sequence, and *background*, which entails containment.

The two levels of the variable place are different and containment. Similarly to temporal sequence, spatial different is used when the situations described by the marked and reference clauses occur at locations that do not stand in a containment relation. Spatial containment is used when the location of one situation includes that of the other one, or when the two situations occur at the same location.

If Mbyá Guaraní SR were similar to Amele SR in its sensitivity to spatio-temporal continuity, we would expect that, everything else being equal, discourse units that describe situations that unfold at different times (time=sequence) or in different places (place=different) should have higher proportions of different markers.

With respect to annotation, the spatial and temporal locations of the situations described by the marked and reference clause were inferred based on the use of spatial and temporal modifiers, the type of events or states described by the marked and reference clause, and contextual cues provided by narratives. Note that because Mbyá Guaraní is a tenseless language, tense inflection cannot serve as a resource in the annotation of spatio-temporal continuity.

### 3.4 Mirativity

Dooley (1992) argues that one of the factors that govern non-canonical uses of SR markers in Mbyá is whether the SR construction describes a sequence of events that unfolds in a predictable fashion. To a certain extent, this factor is captured by the variable polarity, since *contrast* is the only negative rhetorical relation, and counter-expectational *contrast* conveys that the outcome of an event was not as it was expected. In addition, the Mbyá language also has mirative particles at its disposal, whose use in SR constructions gives cues about the speaker’s perception of predictability. Mirativity is a linguistic category whose function is “to mark sentences which report information which is new or surprising to the speaker” (DeLancey 1997). When a mirative particle occurs in the second member of a pair of DUs related by an SR marker, it may convey that the event described by this DU is surprising in the context provided by the first DU. In addition, surprise of a protagonist may be lexically encoded by verbs such as *nhemondyi* (‘be startled’). We expect that, everything else being equal, the presence of mirative particles in the second member of a pair of discourse units related SR will be associated with a higher proportion of different marking.

Every occurrence of SR marker in the corpus was annotated for the presence of a mirative particle or a verb of surprise in the second member of the pair of DUs it relates.8That is to say, using the terminology introduced in Section 3.1, SR constructions were annotated for the presence of a mirative particle in the current DU. The following three mirative particles were considered in our annotation:

(21)

**Table:** 

Mirative particles (definitions from Dooley 2016):^9^

9 The definitions from Dooley (2016) were translated from Brazilian Portuguese into English. Note that Dooley (2016) does not discuss the classification of these particles as mirative. Any errors in this respect are ours.

a.

**Table:** 

*ra’e*: ‘Indicates a discovery, that is, indicates that a fact is verified only in the reported instant.’

b.

**Table:** 

*ri ty*: ‘Indicates surprise about something in the context.’

c.

**Table:** 

*tu*: ‘Indicates intensity and even abruptness.’

We define the variable mirativity, with two levels: TRUE (a mirative particle or verb of surprise is attested in the second DU) and FALSE. The following example illustrates. The second occurrence of the different marker *rã* relates the DUs labelled *π*_*a*_ and *π*_*b*_. Since two mirative markers (*ri ty* and *ra’e*) occur in the second DU, the SR construction that relates *π*_*a*_ and *π*_*b*_ was annotated TRUE for the variable mirativity. Incidentally, we note that SR is used non-canonically in this construction:

(22)

**Table:** 

*O-ma’ẽ*	*rã*	*je*	*,*	*h-endy-pa*	*rei*	*merami*	*rã*	*je*	*,*			
a3-look	df	hsy		b3-glowing-comp	excl	apparently	df	hsy				

${[}_{\pi_{a}}$	*o-i-kuaa*	*pota*	]	***rã***	*je*				
	a3-3-know	try		df_nc_	hsy				

${[}_{\pi_{b}}$	*oo*	*o-vera-pa*	*va’e*	*py*	*ri ty*	*ra’e*	*o-ĩ*	*,*	*kunha*	*va’e*	.	]
	house	a3-shine-compl	nmlz	loc	mir	mir	a3-be		woman	nmlz		
‘She looked, and it seemed that everything was glowing; she tried to understand: she was in a house where everything was shining!’ (Dooley 2011)												

### 3.5 Clause type

The last class of predictors of SR marker choice we considered consists only of one variable, clause_type, which encodes whether an SR construction is full or reduced. There are differences between the grammar of these two construction types that may affect the rate of non-canonical uses of SR markers in each. Indeed, SR markers in full constructions relate clauses associated with a well-defined set of arguments identifiable by grammatical function. By contrast, SR markers in reduced constructions relate a clause to a pronoun that is anaphoric to a preceding discourse unit. The resolution of the pronoun’s antecedent may fail to retrieve the grammatical function of the arguments that introduced the discourse referents mentioned in the antecedent. This in turn may contribute to a higher rate of non-canonical uses, since canonical uses of SR markers track the referential continuity of pivots defined by grammatical function (i.e., subjects).

## 4 Corpus exploration

In this section, we explore the distribution of SR marker types in their canonical and non-canonical uses. Table 2 summarizes our data set. All our predictor variables except additivity and time are significantly associated with marker_type at the *p* < 0.05 significance level. We note in particular that the ratio of different to same markers is significantly higher in the presence of mirative markers than in their absence, and with rhetorical relations of negative polarity as compared to positive polarity.

**Table 2:** Data set summary.

	DF, *N* = 394^a^	SM, *N* = 368	*p*-value^b^
referential_continuity			<0.001
None	165 (100 %)	0 (0 %)	
OO	34 (100 %)	0 (0 %)	
OS.SO	189 (95 %)	10 (5 %)	
SS	6 (2 %)	358 (98 %)	
additivity			0.060
Additive	308 (54 %)	265 (46 %)	
Causal	86 (46 %)	103 (54 %)	
polarity			<0.001
Negative	26 (84 %)	5 (16 %)	
Positive	368 (50 %)	363 (50 %)	
mirativity			<0.001
FALSE	339 (49 %)	352 (51 %)	
TRUE	55 (77 %)	16 (23 %)	
place			0.003
Different	36 (37 %)	61 (63 %)	
Containment	358 (54 %)	307 (46 %)	
time			0.5
Containment	146 (50 %)	147 (50 %)	
Sequence	248 (53 %)	221 (47 %)	
clause_type			<0.001
Full	157 (40 %)	235 (60 %)	
Reduced	237 (64 %)	133 (36 %)	

^a^ *n* (%). ^b^Pearson’s Chi-squared test with Yates continuity correction.

A first generalization about the distribution of canonical and non-canonical SR markers in our corpus can be extracted from the distribution of referential_continuity in Table 2. Note that canonical uses of SR markers correspond either to same markers with an SS value of this variable (in which case the subjects of the marker and reference clauses corefer), or to different markers with a value other than SS (in which case the subjects do not corefer). Focusing on non-canonical uses of SR markers then, we note that all 10 non-canonical uses of same markers in the corpus are attested with an OS.SO value of referential_continuity. Non-canonical same marking is unattested with values OO and none. This distribution is unlikely to be observed under the null hypothesis that non-canonical same markers are equally likely to occur in each of the three non-SS levels of referential_continuity (Exact Multinomial Test: *p* = 5e-5).

The previous observation suggests that SR marker choice is sensitive to finer degrees of referential continuity than the distinction between coreferential subjects versus non-coreferential subjects. More precisely, they suggest that same marking requires a high degree of referential continuity not only in its canonical uses but also in its non-canonical uses, the latter of which are only attested when the subject of the marked or reference clause corefers with one non-subject in the other clause. Consequently, this observation suggests that SR marker choice is always sensitive to referential continuity, both in its canonical uses and in its non-canonical uses. This is problematic for a view of SR that would post a categorical distinction between canonical and non-canonical SR according to which the former would serve a function of reference tracking, while the latter would track non-referential dimensions of thematic continuity. By contrast, it is consistent with the view that canonical and non-canonical uses of SR markers are generated by a single process that is uniformly sensitive to referential continuity.

We now turn to a more detailed examination of the distribution of non-canonical uses of SR marking in the corpus. Table 3 presents the combination of predictor values for which non-canonical same markers are attested in the corpus, together with counts of non-canonical same markers and canonical different markers for each combination.10By definition, canonical same markers and non-canonical different markers are unattested at levels of referential_continuity other than SS. As we already observed, non-canonical same markers are unattested at levels of referential_continuity lower than OS.SO. In addition, we observe that they are only attested with positive rhetorical relations (polarity = positive) and in the absence of mirative markers (mirativity = FALSE). This is consistent with the hypothesis that non-canonical same markers indicate thematic continuity in non-referential dimensions, or at least the absence of discontinuity that would come from rhetorical relations of contrast, or surprise expressed by mirative markers.

**Table 3:** Distribution of non-canonical same markers.

referential continuity	place	time	additivity	polarity	mirativity	clause type	sm noncan.	df can.
OS.SO	Different	Containment	Causal	Positive	FALSE	Reduced	1	0
OS.SO	Different	Sequence	Additive	Positive	FALSE	Reduced	1	10
OS.SO	Containment	Containment	Additive	Positive	FALSE	Reduced	1	14
OS.SO	Containment	Containment	Causal	Positive	FALSE	Reduced	3	3
OS.SO	Containment	Sequence	Additive	Positive	FALSE	Full	1	18
OS.SO	Containment	Sequence	Additive	Positive	FALSE	Reduced	2	72
OS.SO	Containment	Sequence	Causal	Positive	FALSE	Reduced	1	8

Table 4 presents the combination of predictor values for which non-canonical different markers are attested in the corpus, together with counts of non-canonical different markers and canonical same markers for each combination. We observe that non-canonical different markers are only attested with negative rhetorical relations (polarity = negative) or in the presence of mirative markers (mirativity = TRUE). This conforms to the hypothesis that non-canonical different markers indicate thematic discontinuity in non-referential dimensions.

**Table 4:** Distribution of non-canonical different markers.

referential continuity	place	time	additivity	polarity	mirativity	clause type	df noncan.	sm can.
SS	Containment	Containment	Additive	Negative	FALSE	Reduced	1	0
SS	Containment	Containment	Additive	Positive	TRUE	Full	1	3
SS	Containment	Containment	Causal	Negative	FALSE	Full	2	3
SS	Containment	Sequence	Additive	Positive	TRUE	Full	1	5
SS	Containment	Sequence	Additive	Positive	TRUE	Reduced	1	3

Finally, in Tables 3 and 4, we observe that canonical SR markers are attested in all but two contexts in which non-canonical markers are attested in the corpus. Here, “context” refers to a combination of predictor values. To the extent that our predictor variables capture the set of factors that govern SR marker choice, this supports a probabilistic approach to SR marker choice, according to which both same and different markers may have a non-zero probability of use in any given context.11An anonymous reviewer points out that reduced SR constructions seem to favour non-canonical same rather than different marking and asks why this may be the case. We note that clause_type is significantly associated with marker_type overall (canonical and non-canonical uses included), as can be seen in Table 2. We also note that the proportion of different markers overall is *greater* in reduced clauses. While this may seem contradictory at first, remember that both canonical uses of different markers and non-canonical uses of same markers indicate non-coreferential subjects. Remember also that 98 % of all SR markers in the corpus are used canonically, so that the greater proportion of different markers in reduced SR constructions entails a greater proportion of non-coreferential subjects in these constructions. There is indeed a significant association between subject coreference (coreferent vs non-coreferent subjects) and clause_type (Fisher’s exact test: *p* < 0.001). In light of this, we hypothesize that the association between clause_type and marker_type observed both in Table 2 and in Tables 3 and 4 is indirect, subject coreference acting as a mediating variable (note that subject coreference is the same as referential_continuity with all non-SS levels are collapsed into one for simplicity). That is to say, we hypothesize that clause_type influences subject coreference, which in turns influences marker_type. We use the Baron and Kenny method to establish this (Baron and Kenny 1986). First, we regress marker_type on clause_type; clause_type significantly influences marker_type (*p* < 0.001). Secondly, we regress subject coreference on clause_type; clause_type significantly influences subject coreference (*p* < 0.001). Third, we regress marker_type on clause_type controlling for subject coreference; subject coreference significantly influences marker_type (*p* < 0.001) but clause_type does not have a significant effect on marker_type when subject coreference is controlled. Finally, we regress clause_type on marker_type controlling for subject coreference. marker_type does not have a significant effect on clause_type (*p* = 0.14) when subject coreference is controlled. These results support our mediation hypothesis and suggest that the effect is complete. Bootstrap estimation with the Mediation package in R confirms that the indirect effect of subject coreference is significant (*p* < 0.001). The real question, then, is why there is a greater proportion of non-coreferential subject in reduced SR constructions than in full SR constructions. We speculate that this may be an effect of discourse coherence constraints that put more pressure on keeping the sentence topic constant inside than across sentences.


## 5 Modelling switch-reference marker choice

We now proceed to a multi-factorial analysis of SR marker choice. Our hypothesis is that canonical and non-canonical uses of SR markers are generated by the same process, which maps a context (characterized by a combination of predictor values) to a probability of same marker use (or equivalenty of different marker use). In this perspective, the status of SR markers as canonical or non-canonical is neither a parameter of the context nor a class of outcomes in the process that generates SR markers. It is merely an epiphenomenon of SR marker choice. Consequently, neither the predictors of our model nor its outcome variable will encode the distinction between canonical and non-canonical uses. The outcome variable of our model, marker_type, has two levels, same (SM) and different (DF), which stand for the SR markers *vy* and *ramo*∼*rã* respectively. The predictors are the variables discussed in Section 3: referential_continuity, polarity, additivity, mirativity, place, time and clause_type.

The reader may wonder how to evaluate the success of such a model in predicting (non-)canonical uses of SR markers. The performance of our model as a classifier is of limited interest. Indeed, we saw in the previous section that in virtually all contexts where non-canonical uses of SR markers are attested, canonical uses are also attested with a higher frequency. Therefore, a probabilistic classifier that always gives a higher probability to the canonical outcome in these contexts will have better classification performance. Consequently, although we will present standard evaluation metrics to assess the success of our model in predicting same versus different marking, its success in predicting non-canonical uses of SR markers will be assessed by simulating uses of SR markers from the fitted model and comparing them to occurrences observed in our corpus. More precisely, we will use the predictions of our model on holdout data to simulate vectors of same and different markers, and we will ask (i) whether the simulated proportions of non-canonical SR markers match the proportions that are observed in our corpus and (ii) whether the contexts in which non-canonical SR markers are produced in simulations match the contexts in which they are used in the corpus.

The Supplementary Materials present a model of marker_type as a function of pivot coreference only. The interested reader is invited to compare the results presented in the present section to those obtained from this simpler baseline model.

### 5.1 Penalized regression model of SR marker choice

We train a logistic regression model of SR marker choice with the variables discussed in Section 3 as predictors, as specified in formula (23):

(23)

**Table:** 

marker_type ∼ referential_continuity + polarity + additivity + mirativity + place + time + clause_type

When verifying that the assumptions of logistic regression are met for this model, we observe that quasi-complete separation leads to infinite maximum likelihood estimates for several parameters. There is also high multicollinearity between referential_continuity, polarity and mirativity. In order to address these issues, we fit our model with least absolute shrinkage and selection operator (lasso) regularization. Lasso regression shrinks coefficients towards zero by introducing a penalty term scaled by a parameter *λ* (Hastie et al. 2015). In classical lasso regression, a possibly different penalty score is calculated for each predictor. In an extension known as grouped lasso regression, the set of predictors is partitioned in non-overlapping groups, and a penalty score is calculated for each group of predictors. In the case of lasso regression with categorical predictors, this ensures that levels of a categorical predictor coded as different variables are subject to the same penalty (Yuan and Lin 2006).

We fit a grouped lasso model of marker choice with the formula specified in (23) using the R package gglasso (Yi and Hui 2015). Categorical variables are treated using one-hot coding, where each level is coded as a separate variable.12When fitting a Lasso regularized regression model, one-hot coding is preferable to dummy coding, since the latter would encode the reference level of categorical predictors in the intercept, but the lasso does not regularize the intercept. Variables obtained from the same predictors by one-hot coding are grouped together.

The penalty parameter *λ* is estimated by 10-fold cross-validation on the model training set. Because non-canonical uses of SR markers are rare and we need them to be represented both in the training and test data, we cannot train and evaluate the model using a single train-test split. We resort instead to nested cross-validation implemented using the nestedcv package in R (Lewis et al. 2023). We use leave-one-out cross-validation (LOOCV) for the outer loop of nested CV and 10-fold cross-validation for the inner loop. That is to say, for each observation in our data set, we train a model on the 761 remaining observations (this is the outer-loop). The best *λ* parameter for this model is assessed by 10-fold cross-validation on these 761 observations (this is the inner loop). Crucially, when evaluating our model performance in predicting SR marking, we use the model predictions on the holdout data of the outer loop of cross-validation. Note that these predictions are obtained from fitting the model to 762 different training sets, one per fold of LOOCV, although these training sets differ from one another by at most one observation. We report coefficient estimates for a model fitted on the whole data set.

**Figure 2:**
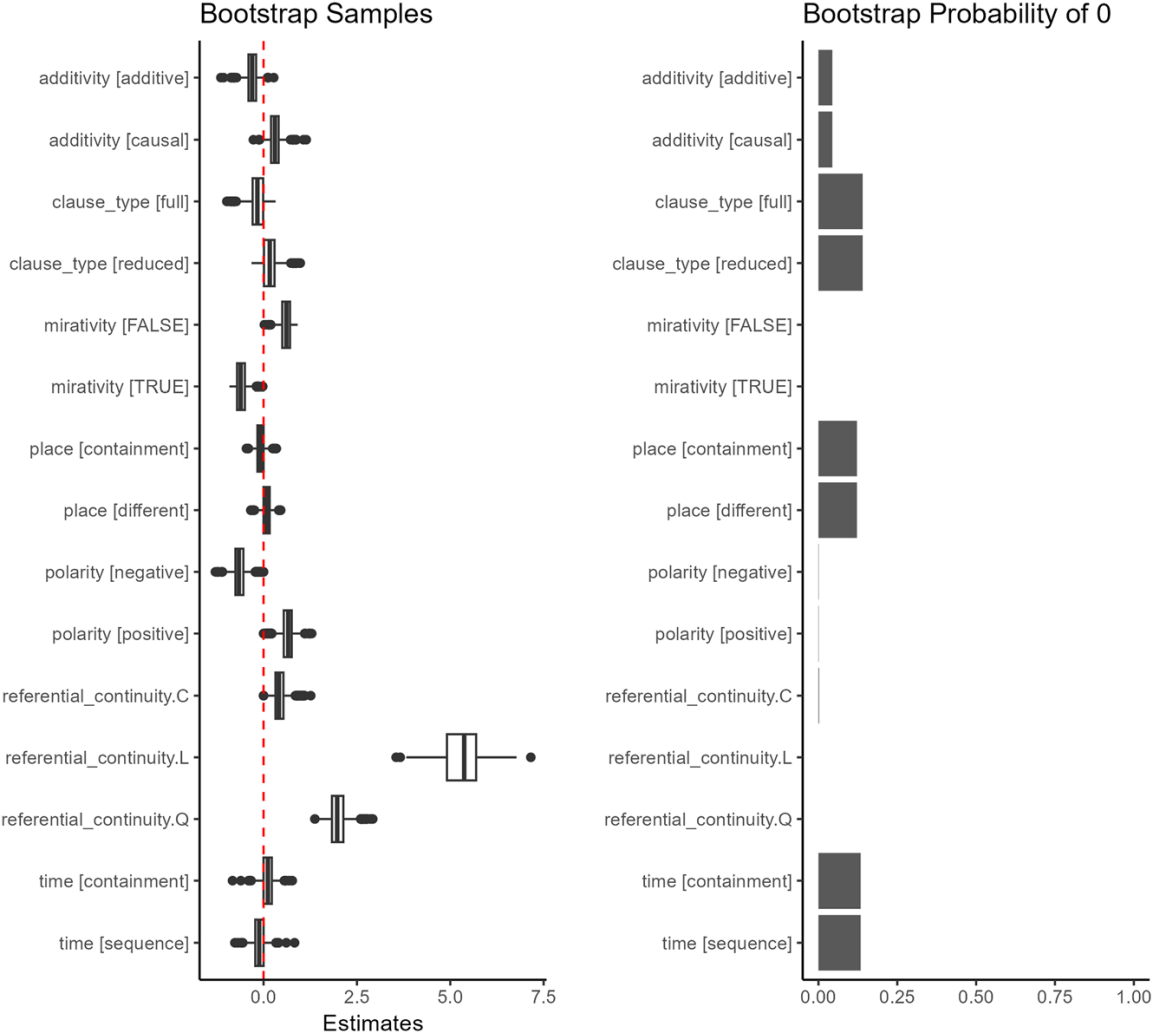
Bootstrap approximation of sampling distribution.

Table 5 presents the coefficient estimates of our model fitted to the complete data set at an optimal value of *λ* = 3.98*e* − 4. Note that we do not provide *p*-values or 95 % confidence intervals.13The reader may wonder whether the interpretation of coefficients is reliable given the presence of high multicollinearity. Multicollinearity inflates standard errors of coefficient estimates in traditional regression models, which results in inaccurate tests of significance. Note that this issue is put aside with Lasso regression, since there is no attempt to provide standard errors given the strong bias that is introduced by regularization. The emphasis of Lasso regression is on prediction rather than on inference. Furthermore, note that the coefficients of our model remain fairly stable across bootstrap samples, as can be seen in Figure 2, which suggests that multicollinearity among the predictors does not result in instability in variable selection in our data set. This is because penalization introduces substantial bias in the estimates, and it is challenging to adjust confidence intervals accordingly (Taylor and Tibshirani 2015). While there are several proposals for statistical inference with penalized regression, this is an open area of research that goes beyond the scope of the present manuscript (for a recent overview, see Kammer et al. 2022). Note also that the ordered factor referential_continuity was coded using polynomial contrasts with linear (L), quadratic (Q) and cubic (C) effects.14These were treated as different groups for regularization.


**Table 5:** Model coefficients at *λ* = 3.98e-4.

	Estimate
additivity [additive]	−0.33
additivity [causal]	0.33
clause_type [full]	−0.15
clause_type [reduced]	0.15
mirativity [FALSE]	0.70
mirativity [TRUE]	−0.70
place [different]	0.16
place [containment]	−0.16
polarity [negative]	−0.71
polarity [positive]	0.71
referential_continuity.L	5.64
referential_continuity.Q	2.10
referential_continuity.C	0.33
time [containment]	0.05
time [sequence]	−0.05

While we cannot provide confidence intervals for the estimates, we can approximate their sampling distribution using bootstrap resampling (Hastie et al. 2015: §6.2). We draw 1,000 samples with replacement of 762 observations from the corpus (as many as there are observations in the corpus). For each sample, we select an optimal value of *λ* by 10-fold cross-validation, and we fit the model on the whole data set at this value of *λ*. Figure 2 displays the distribution of model estimates across the bootstrap samples, as well as the proportion of times each estimate is zero in the bootstrap distribution. We observe that, if we disregard outliers, mirativity, polarity and referential_continuity are never set to zero in the resampling.

It is worth noting that grouped lasso regression performs variable selection in addition to regularization. Indeed, optimization of the objective function for grouped lasso regression may result in setting coefficient estimates to 0 for some predictors. These predictors are effectively excluded from the model. We can observe in Table 5 that no coefficient has been shrunk to 0, therefore all variables have been retained.

We assess the model performance on holdout data of the LOOCV process. The model has excellent discrimination, with a C-index of 0.99.15For binary outcomes, the C-index is a measure of the probability that a randomly selected observation that has a positive value of the outcome variable has a higher predicted probability of positive outcome than a randomly selected observation that has a negative value of the outcome variable. The C-index is equivalent to the area under the receiver operating characteristic curve in the case of binary outcomes (Harrell 2015: §10.8). This is not surprising since 98 % of all SR markers in the corpus are canonical and can therefore be perfectly predicted by referential_continuity. For comparison, a logistic regression model of marker_type with pivot coreference as its unique predictor16Where pivot coreference has two levels: coreferential subjects versus non-coreferential subjects. has a C-index of 0.96, and an intercept-only model has a C-index of 0.5.

### 5.2 Analysis of model predictions

We wish to know (i) whether the frequencies of non-canonical same and different markers in data generated by the model match the frequencies observed in the corpus and (ii) whether the contexts in which the model tends to generate non-canonical same and different markers match the contexts in which they are observed in the corpus.

#### 5.2.1 Predicted probabilities of same marking across contexts

**Figure 3:**
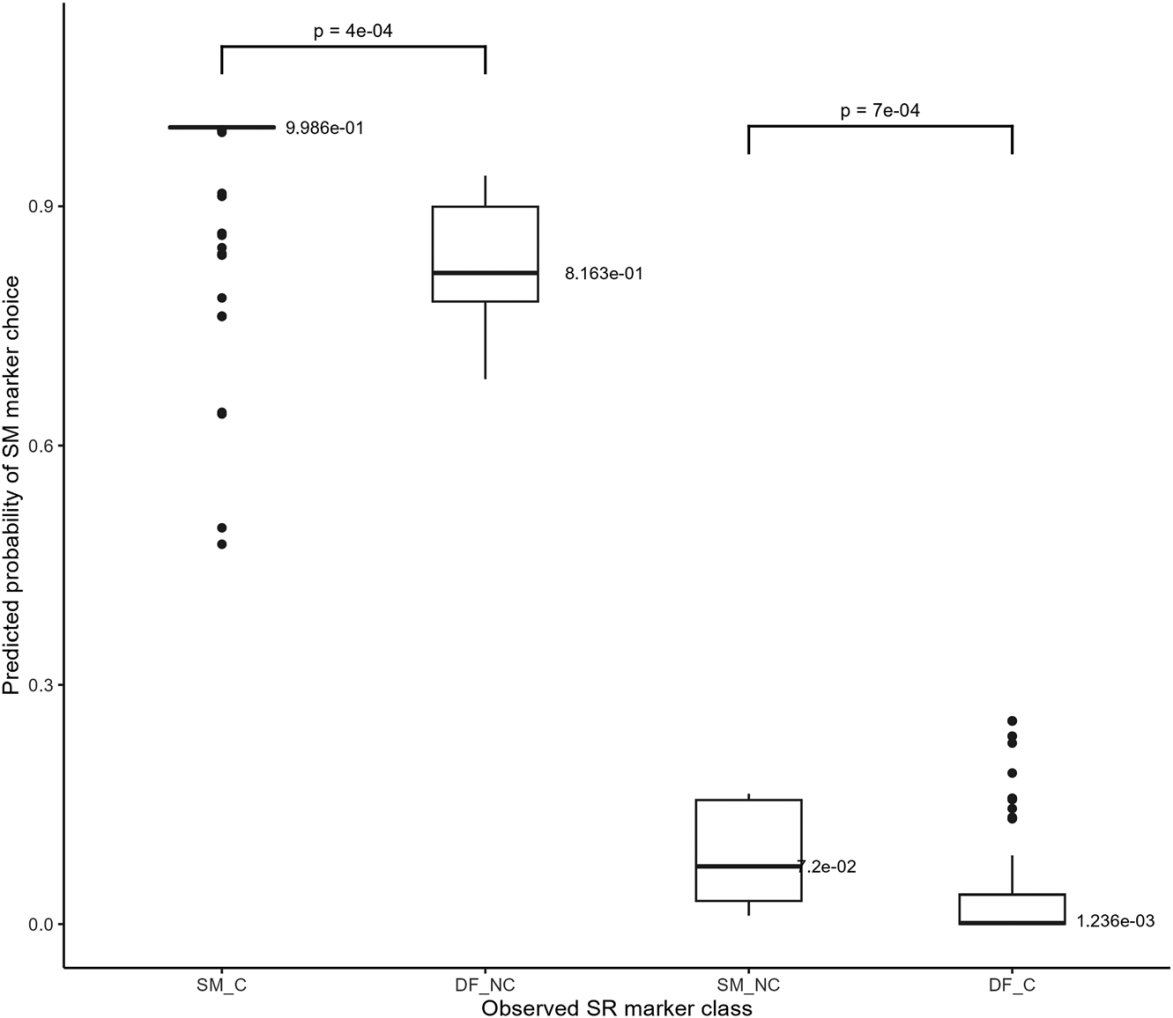
Predicted probabilities of same marker choice for different SR marker classes.

Before we address these questions directly, let us compare the predicted probabilities of same marker choice for different classes of SR markers observed in our corpus. The predicted probabilities are obtained from holdout data in the LOOCV process. As a result, a single probability of same marking is predicted for each observation in the corpus. We can therefore group these predicted probabilities according to the type of SR markers that are attested in the corpus for their matching observations, and whether these markers are used canonically or not in the corpus. This results in four classes of predictions, each of which corresponds to a class of SR makers attested in the corpus: canonical same markers (SM_C), non-canonical different markers (DF_NC), non-canonical same marker (SM_NC) and canonical different markers (DF_C). Figure 3 displays the probabilities of same marker choice predicted by the model for each class of observations.

Note that observations for which a canonical same marker was attested in the corpus are similar to observations for which a non-canonical different marker was attested, insofar as the value of referential continuity is SS for both types of observations. These observations are grouped in the two leftmost box plots in Figure 3. Likewise, observations for which a non-canonical same marker was attested in the corpus are similar to observations for which a canonical different marker was attested, insofar as the value of referential_continuity is not SS for either type of observations. These observations are grouped in the two rightmost box plots.

Figure 3 shows that the probabilities of same marking predicted by the model are significantly higher with observations for which a canonical same marker is attested in the corpus, than with observations for which a non-canonical different marker is attested (two-sample permutation test for the mean: *p* = 4e-04). In other words, the model correctly predicts that the use of a different marker is significantly more probable with observations for which non-canonical different markers are actually attested than with observations for which canonical same markers are attested.

Likewise, the probabilities of same marking predicted by the model are significantly lower with observations for which a canonical different marker is attested in the corpus, than with observations for which a non-canonical same marker is attested (two-sample permutation test for the mean: *p* = 7e-04). In other words, the model correctly predicts that the use of a same marker is significantly more probable with observations for which non-canonical same markers are actually attested than with observations for which canonical different markers are attested.

#### 5.2.2 Frequency of non-canonical SR markers

In order to investigate the model’s predictions more in depth, we use our model to simulate new observations, and we explore the frequency of non-canonical SR markers in these simulations, as well as the contexts in which non-canonical SR markers are generated. To do so, we gather the probabilities of same marking predicted by the model17Remember that these are predictions made on holdout data in the leave-one out cross-validation process, therefore the observation for which each prediction is made was not included in the corresponding training set. into a vector of 762 probabilities, one for each observation in our corpus. For each element *p* in this vector, we generate an SR marker randomly, with a probability *p* of generating a same marker, and a probability 1 − *p* of generating a different marker. This results in a vector of 762 SR markers. We repeat the process 1,000 times, for a total of 1,000 vectors of 762 SR markers. Note that each SR marker generated in this way is associated with a combination of predictor values. We can therefore determine whether an SR marker generated as part of this simulation is an instance of canonical or non-canonical SR marking: non-canonical markers are same markers that were generated with a referential_continuity value other than SS, or different markers that were generated with an SS value of referential_continuity.

**Figure 4:**
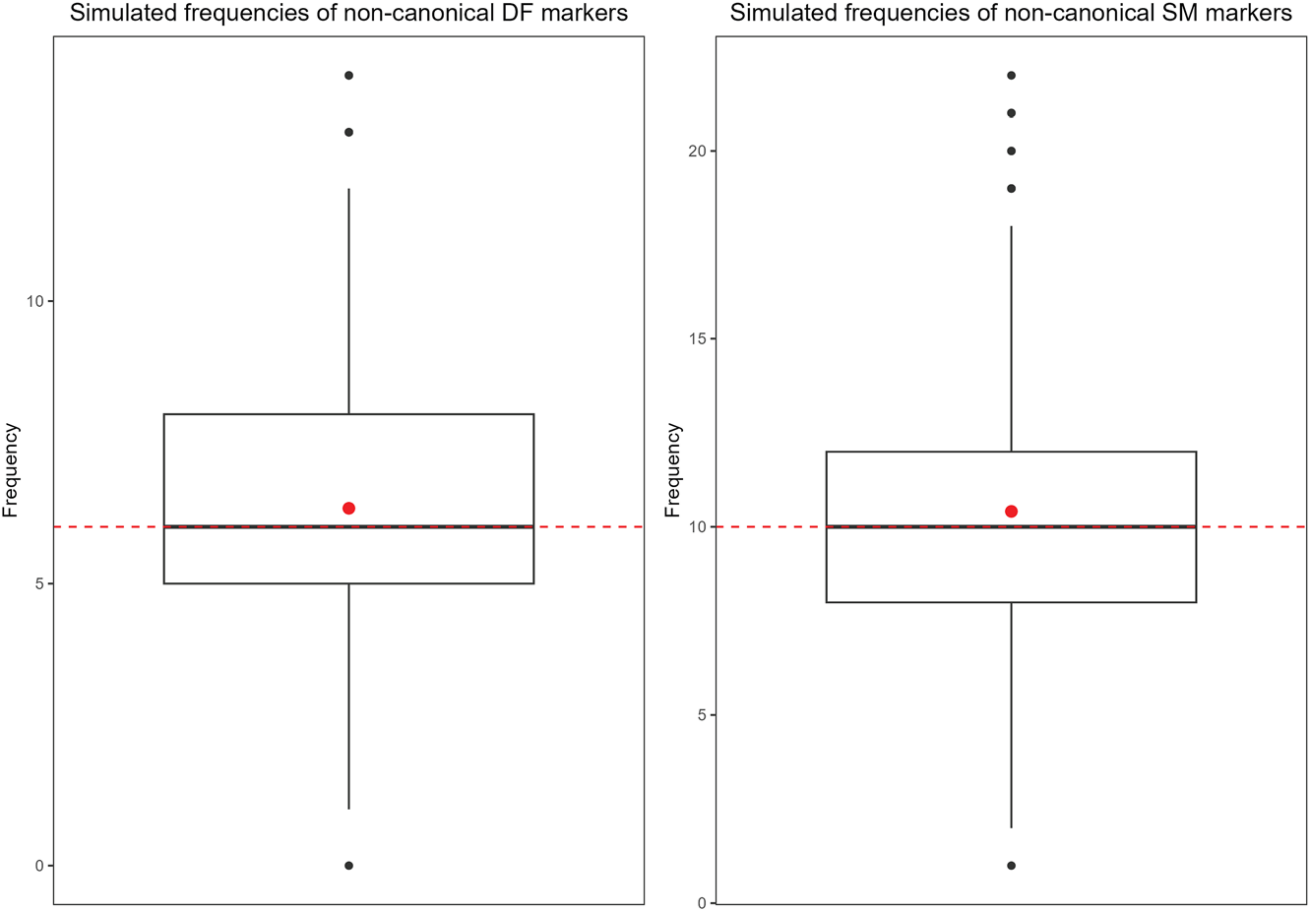
Frequencies of non-canonical SR markers in 1,000 simulated data sets.

Figure 4 displays the frequencies of non-canonical same and different markers in the simulated data sets, with the mean represented by a red dot in each plot. The frequency of non-canonical markers attested in the corpus is superimposed to each plot as a dashed red line. For both different and same markers, we observe that the median frequency of non-canonical markers across simulated data sets is identical to the frequency observed in the corpus. This corresponds to 6 non-canonical different markers and 10 non-canonical same markers. In addition, the frequency of non-canonical different markers observed in the corpus is only −0.15 standard deviations away from the mean of simulated frequencies (one-sample permutation test for the mean: *p* < 0.0001). Likewise, the frequency of non-canonical same markers observed in the corpus is only −0.15 standard deviations away from the mean of simulated frequencies (*p* < 0.0001). We conclude that the frequencies of non-canonical SR markers in data sets generated by the model are similar on average to the frequencies observed in the corpus.

#### 5.2.3 Contexts of occurrence of non-canonical SR markers

Next, we ask whether the contexts in which the model generates non-canonical SR markers are similar to the contexts in which SR markers are used non-canonically in the corpus. To do so, we merge the 1,000 data sets generated using the procedure described in Section 5.2.2 into a single data set with 762,000 simulated observations of SR markers. We then fit a classification tree model of SR marker choice to this data set, as specified in formula (24). Note that our goal in fitting this model is to explore the structure of the simulated data set, rather than to make predictions about new observations.

(24)

**Table:** 

marker_type ∼ referential_continuity + polarity + additivity +mirativity + place + time + clause_type.

**Figure 5:**
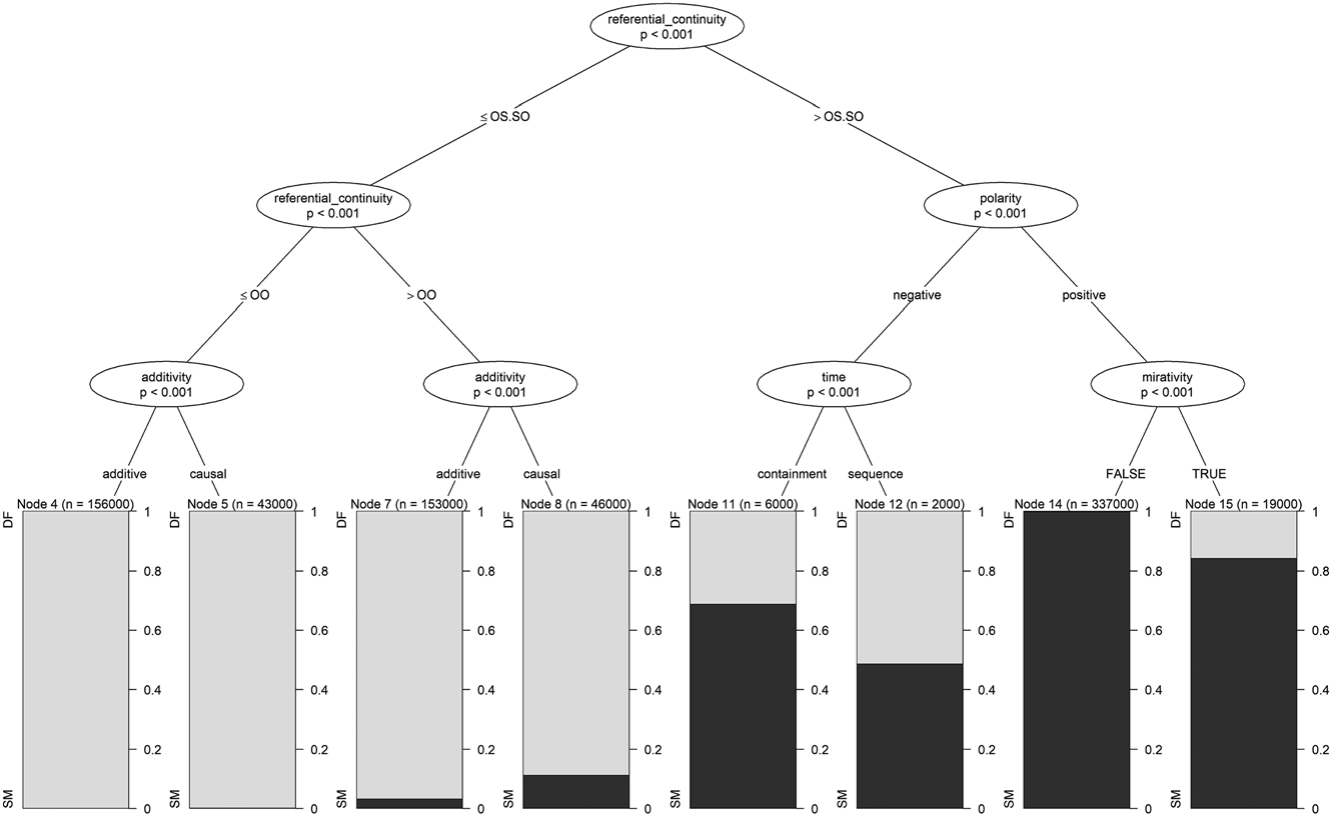
Classification tree of marker_choice in simulated data.

The classification tree is fitted using the ctree function of the partykit package in R (Hothorn and Zeileis 2015). The model is fitted by choosing a predictor to split the data set in two subsets. The same process is repeated on each subset, until a stopping criterion is met. At each step of the process, the predictor that is used to perform the split is chosen by performing a series of permutation tests of independence between each predictor and the outcome variable. The predictor with the most significant association to the outcome variable is selected.18If the predictor has more than two levels, a similar procedure is applied to all possible binary splits of the outcome variable along the predictor, and the split with the most significant association to the outcome variable is chosen. The process stops once no predictor is significantly associated with the outcome variable at a level of significance *α*. Other stopping criteria can be specified, such as maximal tree depth or minimum number of observations in subsets at each split. We set the level for predictor selection at *α* = 0.05, and the maximal tree depth at 3.19Trees with more depths would have terminal nodes that are subsets of the terminal nodes displayed in Figure 5. While such trees might perform differently for classifying new observations, their added complexity is not needed for the exploratory purpose of this section.


The fitted tree is displayed in Figure 5. At the root of the tree, referential_continuity splits the whole data set into two groups: observations with an SS value of this predictor are gathered in the subset to the right20
SS is the only level *l* of referential_continuity such that *l* > OS.SO. and all other observations are gathered in the subset to the left. The left subset therefore contains canonical different markers and non-canonical same markers, while the right subset contains canonical same markers and non-canonical different markers. Focusing on the left subset first, we observe that non-canonical same markers are only attested with an OS.SO value of referential_continuity, and are more frequent with causal rhetorical relations. Moving on to the right subset, we observe that non-canonical different markers are only attested with negative rhetorical relations or in the presence of mirative markers.

By comparing this classification tree with Tables 3 and 4 of Section 4, we observe that the contexts in which the model generates non-canonical same and different markers correspond to those in which these markers are attested non-canonically in the corpus. In particular, non-canonical uses of same markers are only attested with a high degree of referential continuity, and non-canonical different markers are only attested with negative rhetorical relations or in the presence of mirative markers.

In sum, it appears that our model of marker_type, although blind to the difference between canonical and non-canonical uses of SR markers, generates non-canonical uses with adequate frequency in adequate contexts.

## 6 Discussion

The results of the previous section suggest that the distinction between canonical and non-canonical uses of SR markers can be analyzed as a side effect of a probabilistic and multifactorial process of SR marker choice that is itself blind to canonicity. More precisely, it appears that SR marker choice is sensitive to a multiplicity of factors besides referential continuity, including notably mirativity and the polarity of rhetorical relations, which manifest non-referential aspects of thematic continuity. While referential continuity appears to have the highest weight in determining SR marker choice, non-referential aspects of thematic continuity also affect this process, albeit with a lesser weight. The competition between these factors results in a non-negligible probability of non-canonical uses of SR markers in certain contexts. One virtue of such a probabilistic model of SR marker choice is that it allows us to formulate predictions not only about the contexts in which non-canonical uses are likely to be attested, but also about the expected frequencies of non-canonical uses of SR markers in different contexts.

Our analysis fits within a broader tradition of regression- and corpus-based research on syntactic alternations well illustrated by Bresnan et al.’s (2007) work on the English dative alternation (see also Gries 2017 for a review of research in this tradition). The probabilistic and multifactorial models produced in such studies can be readily interpreted within usage-based theories of grammar, which posit that linguistic representations emerge from repeated exposure to particular instances of constructions, and retain specific features of these instances (Bybee 2006). In the present study, the SR markers *vy*, *ramo* and *rã* may be seen as constructions whose representations include features that instantiate the independent variables of our model, such as referential continuity, mirativity and polarity. Crucially, the strength of association between such features and the phonetic form of the markers varies with the frequency of occurrence of the markers in the different contexts in which the speaker was exposed to their use (Bybee 2006: §13). It is these variable associations of form and meaning that determine the probabilities of canonical and non-canonical uses of SR markers in production.

Our analysis contrasts with categorical accounts of SR marker choice, according to which same and different markers are associated with necessary and sufficient conditions of use. One example of such an account is Stirling’s (1993) analysis of SR marker choice in Eastern Pomo, Lenakel and Amele. Here, we illustrate with Stirling’s analysis of Lenakel. Stirling (1993: 152) observes that Lenakel speakers use same subject (SS) markers unless the marked and reference clauses have different tenses or the reference of the main protagonist changes across clauses, in which case a different subject (DS) marker is used:

(25)

**Table:** 

For Lenakel:	
if tense changes, use DS;	
otherwise, if reference changes, use DS;	
otherwise, if tense and reference stay the same, use SS.	(Stirling 1993: 152)

Stirling argues that SR markers indicate agreement between parameters of the events described by the marked and reference clauses. In the case of Lenakel, a difference between future and non-future tense is argued to indicate that the two events differ in actuality. Consequently, if the marked and reference clauses describe events *a*_*i*_ and *a*_*j*_, SS marking indicates that Protagonist(*a*_*i*_) = Protagonist(*a*_*j*_) & Actuality(*a*_*i*_) = Actuality(*a*_*j*_), while DS marking indicates that Protagonist(*a*_*i*_) ≠ Protagonist(*a*_*j*_) ∨ Actuality(*a*_*i*_) ≠ Actuality(*a*_*j*_).

Our argument against categorical analyses of SR marker choice in Mbyá was that canonical uses of SR markers are attested in virtually all contexts where non-canonical uses are attested. Therefore, there is no combination of predictor values that act as necessary and sufficient conditions for same or different marker choice. This supports a probabilistic approach to SR marker choice, which attempts to model the relative frequencies of canonical and non-canonical uses of SR markers in different contexts.

An analysis of SR related to ours was defended in Pustet’s (2013) study of clause linkers in Lakota.21Note that Pustet (2013) does not use the term “Switch Reference” to refer to a class of expressions, but rather to the reference tracking function of clause linkers. What she calls “Switch Reference” corresponds roughly to our referential_continuity predictor. Pustet argues that the function of Lakota clause linkers is to indicate higher or lower degrees of discourse cohesion (which corresponds to what we called thematic continuity in the present manuscript). Furthermore, she argues that discourse cohesion in Lakota should be decomposed into four parameters: “Switch Reference” (i.e., referential continuity across pivots), “probability” (i.e., whether the second clause in a linkage construction expresses a deviation from the expected course of events), temporal cohesion and contrast. A limitation of Pustet’s study is that while it offers a quantitative analysis of the association between clause linker choice and these different parameters, it does not present a multivariate model of clause linker choice but relies on a collection of univariate models instead. In the spirit of Pustet’s (2013) study, we advocated for a multifactorial analysis of SR marker choice that acknowledges both the referential and the non-referential dimensions of thematic continuity. A further contribution of the present manuscript is the implementation of such an analysis as a probabilistic model of SR marker choice, and the discussion of strategies for evaluating the extent to which such a model captures both canonical and non-canonical uses of SR markers.

## Supplementary Material

Supplementary Material

## Glossing abbreviations for Mbyá examples

a1sg: first person singular active
abl: ablative
ana: anaphoric pronoun
asp: aspect
attn: attention
b1sg: first person singular inactive inflection
bdy: information structure boundary
cf: conterfactual
conv: converb
comit: comitative causative
comp: completive marker
cont: continuative
dat: dative
dim: diminutive
dir: directional
df: different switch-reference marker
excl: exclusive
frust: frustrative
fut: future
hab: habitual
hsy: hearsay evidential
int: intensifier
loc: locative
man: manner
mir: mirative
neg: negation
nmlz: nominalizer
npossd: non-possessed
obj: object marker
q: question particle
pl: plural
r: linking morpheme
sm: same Switch-Reference marker
src: source
seq: sequential

## References

[j_cllt-2024-0015_ref_001] Asher Nicholas, Lascarides Alex (2003). *Logics of conversation*.

[j_cllt-2024-0015_ref_002] Baron Reuben M., Kenny David A. (1986). The moderator-mediator variable distinction in social psychological research: Conceptual, strategic, and statistical considerations. *Journal of Personality and Social Psychology*.

[j_cllt-2024-0015_ref_003] Behrens Bergljot, Fabricius-Hansen Cathrine, Shu Dingfand, Turner Ken (2010). The relation accompanying circumstance across languages: Conflict between linguistic expression and discourse subordination?. *Contrasting meaning in languages of the east and west*.

[j_cllt-2024-0015_ref_004] Behrens Bergljot, Fabricius-Hansen Cathrine, Solfjeld Kåre, Cathrine Kåre Solfjeld, Haug Dag (2012). Competing structues: The discourse perspective. *Big events, small clauses*.

[j_cllt-2024-0015_ref_005] Bresnan Joan, Cueni Anna, Nikitina Tatiana, Baayen Harald, Bouma Gerlof, Maria Krämer Irene, Zwarts Joost (2007). Predicting the dative alternation. *Cognitive foundations of interpretation*.

[j_cllt-2024-0015_ref_006] Bybee Joan (2006). From usage to grammar: The mind’s response to repetition. *Language*.

[j_cllt-2024-0015_ref_007] DeLancey Scott (1997). Mirativity: The grammatical marking of unexpected information. *Linguistic Typology*.

[j_cllt-2024-0015_ref_008] Dooley Robert A. (1989). Switch reference in Mbyá Guaraní: A fair-weather phenomenon. *Work Papers of the Summer Institute of Linguistics, University of North Dakota Session*.

[j_cllt-2024-0015_ref_009] Dooley Robert A., Hwang Shin Ja J., Merrified William R. (1992). When switch reference moves to discourse: Developmental markers in Mbyá Guarani. *Language in context: Essays for Robert E. Longacre*.

[j_cllt-2024-0015_ref_010] Dooley Robert A., Loos Eugene (1999). A noncategorial approach to coherence relations: Switch reference constructions in Mbyá Guaraní. *Logical relations in discourse*.

[j_cllt-2024-0015_ref_011] Dooley Robert A. (2011). Mbyá Guaraní collection of Robert Dooley. ..

[j_cllt-2024-0015_ref_012] Dooley Robert A. (2015). *Léxico Guarani, dialeto Mbyá: Introdução* [Guarani lexicon, Mbya dialect: introduction].

[j_cllt-2024-0015_ref_013] Dooley Robert A. (2016). *Léxico Guarani, dialeto Mbyá* [Guarani lexicon, Mbya dialect].

[j_cllt-2024-0015_ref_014] Givón Talmy (2001). *Syntax: An introduction*.

[j_cllt-2024-0015_ref_015] Gries Stefan Th. (2017). Syntactic alternation research: Taking stock and some suggestions for the future. *Belgian Journal of Linguistics*.

[j_cllt-2024-0015_ref_016] Grosz Barbara, Sidner Candace L. (1986). Attention, intentions, and the structure of discourse. *Computational Linguistics*.

[j_cllt-2024-0015_ref_017] Grosz Barbara, Joshi Aravind K., Weinstein Scott (1995). Centering: A framework for modeling the local coherence of discourse. *Computational Linguistics*.

[j_cllt-2024-0015_ref_018] Guérin Valérie (2019). *Bridging constructions*.

[j_cllt-2024-0015_ref_019] Guillaume Antoine, van Gijn Rik, Haude Katharina, Muysken Pieter (2011). Subordinate clauses, switch-reference, and tail-head linkage in Cavineña narratives. *Subordination in native South American languages*.

[j_cllt-2024-0015_ref_020] Haiman John, Munro Pamela, Haiman John, Munro Pamela (1983). Introduction. *Switch-reference and universal grammar*.

[j_cllt-2024-0015_ref_021] Harrell Frank E. (2015). *Regression modeling strategies*.

[j_cllt-2024-0015_ref_022] Hastie Trevor, Tibshirani Robert, Wainwright Martin (2015). *Statistical learning with sparsity*.

[j_cllt-2024-0015_ref_023] Hobbs Jerry R. (1985). *On the coherence and structure of discourse*.

[j_cllt-2024-0015_ref_024] Hothorn Torsten, Zeileis Achim (2015). partykit: A modular toolkit for recursive partytioning in R. *Journal of Machine Learning Research*.

[j_cllt-2024-0015_ref_025] Kammer Michael, Dunkler Daniela, Michiels Stefan, Heinze Georg (2022). Evaluating methods for lasso selective inference in biomedical research: A comparative simulation study. *BMC Medical Research Methodology*.

[j_cllt-2024-0015_ref_026] Kamp Hans, Reyle Uwe (1993). *From discourse to logic*.

[j_cllt-2024-0015_ref_027] Ladeira Maria Inês, Ricardo Fany Pantaleoni (2018). Guarani mbya. *Povos indígenas no brasil* [Indigenous Peoples in Brazil].

[j_cllt-2024-0015_ref_028] Lewis Myles J., Spiliopoulou Athina, Goldmann Katriona, Pitzalis Costantino, McKeigue Paul, Barnes Michael R. (2023). nestedcv: An R package for fast implementation of nested cross-validation with embedded feature selection designed for transcriptomics and high-dimensional data. *Bioinformatics Advances*.

[j_cllt-2024-0015_ref_029] Mann William C., Thompson Sandra A. (1988). Rhetorical structure theory: Toward a functional theory of text organization. *Text*.

[j_cllt-2024-0015_ref_030] McKenzie Andrew (2014). On the emergence of discourse functions from semantics and pragmatics. ..

[j_cllt-2024-0015_ref_031] Mithun Marianne (1993). Switch-reference: Clause combining in central pomo. *International Journal of American Linguistics*.

[j_cllt-2024-0015_ref_032] Mithun Marianne, Epps Patience, Law Danny, Pat-El Na’ama (2021). Switch-reference: Clause combining in central pomo. *Historical linguistics and endangered languages*.

[j_cllt-2024-0015_ref_033] Muller Philippe, Vergez-Couret Marianne, Prévot Laurent, Asher Nicholas, Farah Benamara, Bras Myriam, Le Draoulec Anne, Vieu Laure (2012). Manuel d’annotation en relations de discours du projet ANNODIS. ..

[j_cllt-2024-0015_ref_034] Pustet Regina (2013). Switch-reference or coordination? A quantitative approach to clause linkage in Lakota. *International Journal of American Linguistics*.

[j_cllt-2024-0015_ref_035] Reese Brian, Hunter Julie, Asher Nicholas, Denis Pascal, Baldridge Jason (2007). *Reference manual for the analysis and annotation of rhetorical structure*.

[j_cllt-2024-0015_ref_036] Roberts John (1988). Amele switch-reference and the theory of grammar. *Linguistic Inquiry*.

[j_cllt-2024-0015_ref_037] Roberts John, Aikhenvald Alexandra Y. (2017). A typology of switch reference. *The Cambridge handbook of linguistic typology*.

[j_cllt-2024-0015_ref_038] Sanders Ted J. M., Demberg Vera, Hoek Jet, Scholman Merel C. J., Asr Fatemeh Torabi, Zufferey Sandrine, Evers-Vermeul Jacqueline (2021). Unifying dimensions in coherence relations: How various annotation frameworks are related. *Corpus Linguistics and Linguistic Theory*.

[j_cllt-2024-0015_ref_039] Stirling Lesley (1993). *Switch-reference and discourse representation*.

[j_cllt-2024-0015_ref_040] Taylor Jonathan, Tibshirani Robert (2015). Statistical learning and selective inference. *Proceedings of the National Academy of Sciences*.

[j_cllt-2024-0015_ref_041] Thomas Guillaume, Antono Gregory, Bradford Laurestine, Kiss Angelika, Winkelman Darragh. (2021). Switch-reference and its role in referential choice in mbyá Guaraní narratives. *Corpus Linguistics and Linguistic Theory*.

[j_cllt-2024-0015_ref_042] van Gijn Rik (2012). Switch-attention (aka switch-reference) in South-American temporal clauses: Facilitating oral transmission. *Linguistic Discovery*.

[j_cllt-2024-0015_ref_043] van Gijn Rik, van Gijn Rik, Hammond Jeremmy (2016a). Switch-reference: An overview. *Switch-reference 2.0*.

[j_cllt-2024-0015_ref_044] van Gijn Rik, van Gijn Rik, Hammond Jeremmy (2016b). Switch-reference in western South-America. *Switch-reference 2.0*.

[j_cllt-2024-0015_ref_045] Veríssimo Tupã A. (2002a). *Opa mba’e re nhanhembo’e aguã 1* [Let us learn about everything 1].

[j_cllt-2024-0015_ref_046] Veríssimo Tupã A. (2002b). *Opa mba’e re nhanhembo’e aguã 2* [Let us learn about everything 2].

[j_cllt-2024-0015_ref_047] Watkins Laurel J. (1993). The discourse functions of Kiowa switch-reference. *International Journal of American Linguistics*.

[j_cllt-2024-0015_ref_048] Yi Yang, Hui Zou (2015). A fast unified algorithm for solving group-lasso penalized learning problems. *Statistics and Computing*.

[j_cllt-2024-0015_ref_049] Yuan Ming, Lin Yi (2006). Model selection and estimation in regression with grouped variables. *Journal of the Royal Statistical Society: Series B (Statistical Methodology)*.

