# Supplementary material for: A multivariate analysis of canonical and non-canonical uses of switch-reference markers in Mbyá narratives
Source: Corpus Linguist Linguist Theory. 2024 Dec 5;22(1):57–91. doi: 10.1515/cllt-2024-0015 (PMC12919634; doi:10.1515/cllt-2024-0015)
Supplement: Supplementary file 1 — Supplementary Material [file j_cllt-2024-0015_suppl_001.pdf]

Supplementary materials: baseline model of SR marker choice

As a baseline, we use a logistic regression model of `marker_type` with pivot coreference as its unique predictor. We fit this model to our data set using LOOCV. The model has a C-index of 0.96.

(1) $\texttt{marker\_type} \sim \texttt{coreference}$

Figure 1 presents the predicted probabilities of SAME marker choice for different classes of SR markers observed in our corpus. We observe that the baseline model fails to predict significantly different mean probabilities of SAME marking for contexts in which non-canonical markers are attested in the corpus, compared to contexts in which canonical markers are attested. This is expected, since the model does not incorporate predictors that would allow it to tease these contexts apart during training.

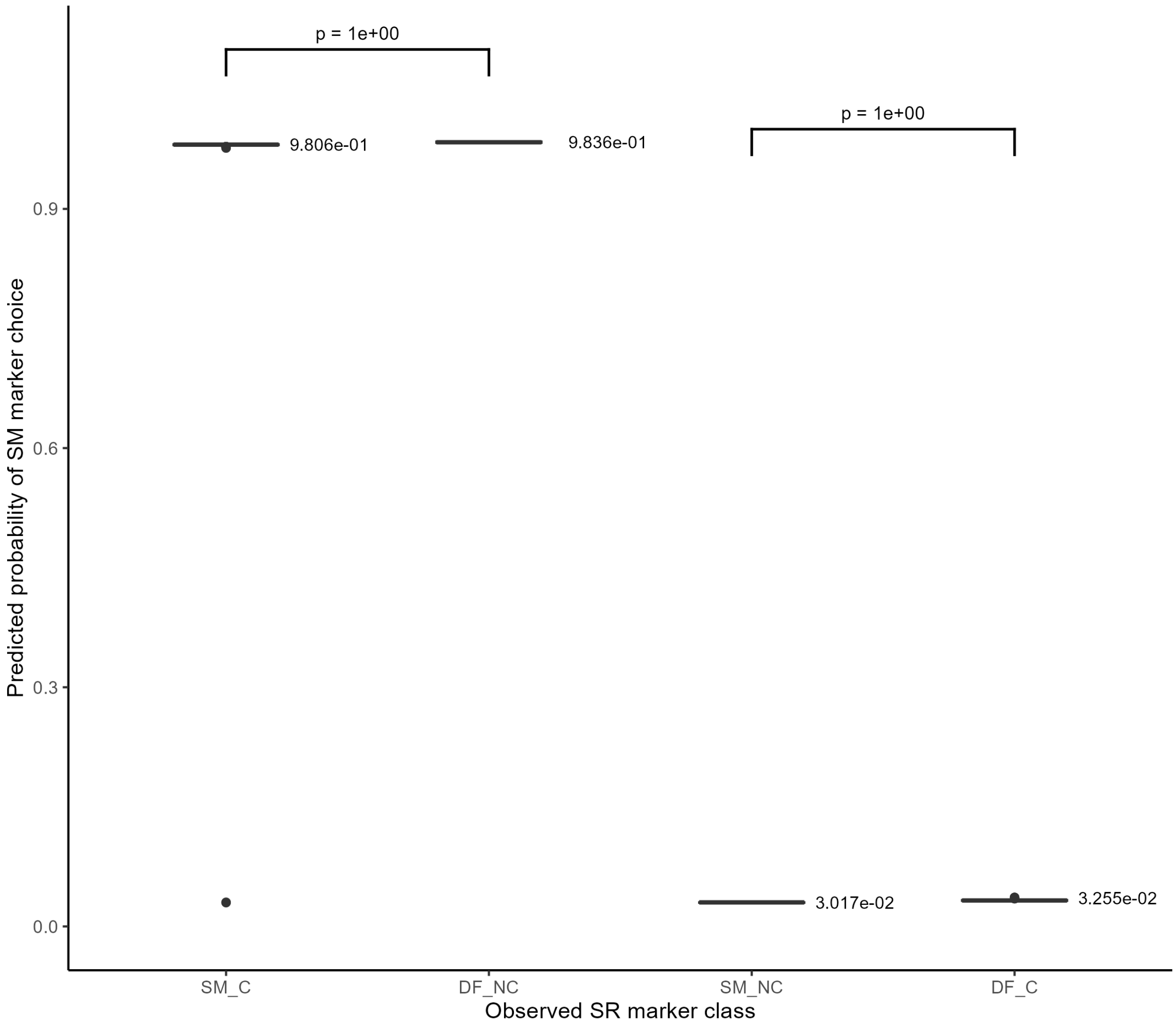

Figure 1: Predicted probabilities of SAME marker choice, subject coreference model

Figure 2 shows the frequencies of non-canonical SAME and DIFFERENT markers in 1,000 simulated data sets generated from the probabilities of SAME marking predicted by the baseline model on holdout data of the LOOCV process. We observe that the median frequencies of non-canonical DIFFERENT and SAME markers in the simulated data sets (9 and 13, respectively, represented by dashed red lines in figure 2) are higher than the frequencies observed in the corpus (6 and 10, respectively). Furthermore, the mean frequency of non-canonical DIFFERENT markers observed in the corpus is -1.1 standard deviations away from the mean of simulated frequencies ($p < 0.0001$), and the the mean frequency of non-canonical SAME markers observed in the corpus is -0.9 standard deviations away from the mean of simulated frequencies ($p < 0.0001$).

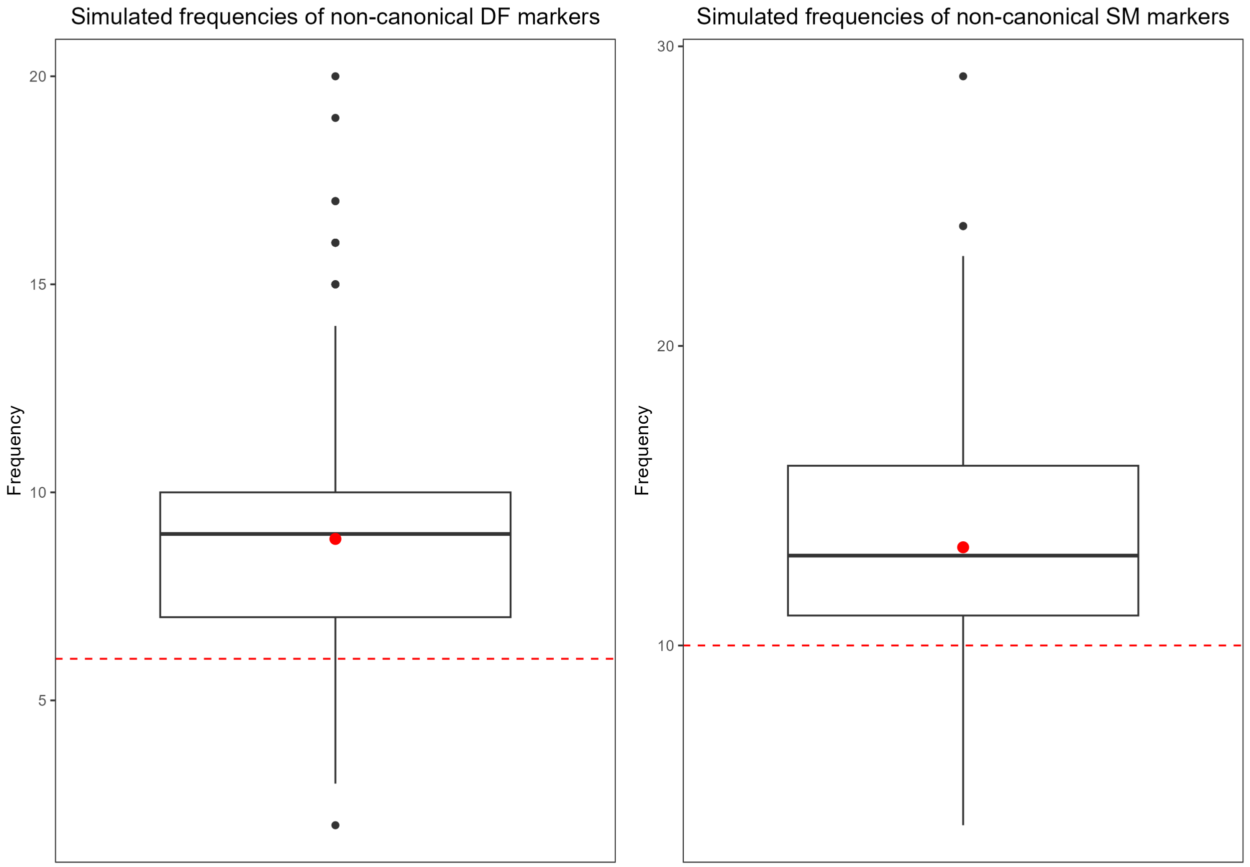

Figure 2: Frequencies of non-canonical SR markers in data sets simulated from coreference model

In sum, the data sets simulated from the predictions of the coreference-only model have a higher frequency of non-canonical markers than the corpus data. This can be explained by the fact that the presence of non-canonical uses of SR markers in the training data of this model results in shifting the predicted probabilities of SAME and DIFFERENT marker choice closer to the mean across the board, even in contexts where SR markers are used canonically in the corpus. Since these contexts are over-represented, this results in a higher frequency of non-canonical uses of SR markers in the simulated data overall.

Finally, we ask whether the contexts in which the baseline model generates non-canonical SR markers are similar to the contexts in which SR markers are used non-canonically in the corpus. To do so, fit a classification tree model of SR marker choice to the simulated data obtained from the

baseline model:

(2) `marker_type` ∼ `referential_continuity` + `polarity` + `additivity` + `mirativity` + `place` + `time` + `clause_type`.

The classification tree is fitted using the `ctree` function of the `partykit` package in `R` (Hothorn and Zeileis, 2015). Figure 3 shows a classification tree fitted with the same formula and parameters on 762,000 simulated observations generated using the probabilities predicted by the coreference-only model on the holdout data of the LOOCV process:

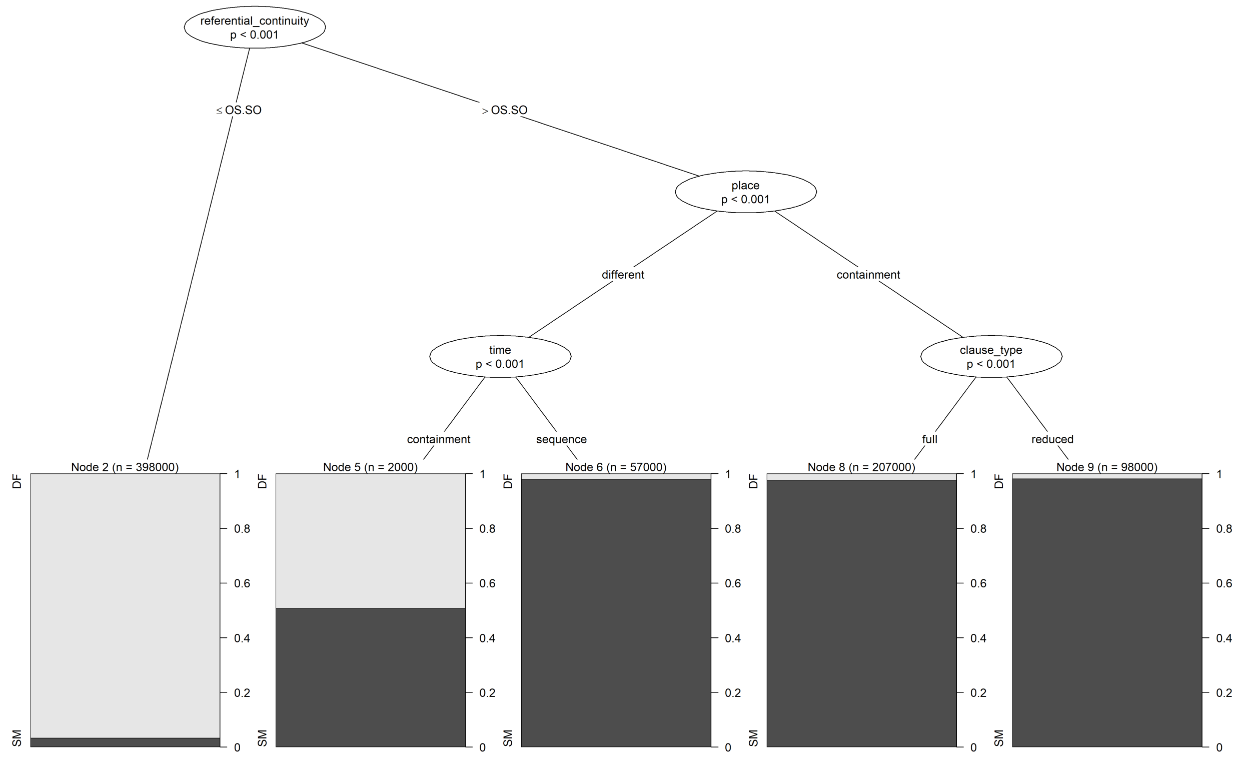

Figure 3: Classification tree of `marker_choice` in data simulated from coreference model

At the root of the tree, `referential_continuity` splits the data into a group of observations with coreferential pivots on the right and another group of observations with non-coreferential pivots on the left. No further predictor is significantly associated with `marker_type` in the latter group. In the former, observations are further split by `place` and then by `time` and `clause_type`. This shows that the contexts in which the baseline model generates non-canonical uses of SR markers fail to match the contexts in which such markers are attested in the corpus. This is expected, since this model cannot learn to adjust probabilities of SAME marker choice across the relevant contexts, given that its only predictor is pivot `coreference`.

References

Hothorn, T. and Zeileis, A. (2015). partykit: A modular toolkit for recursive partytioning in r. *Journal of Machine Learning Research*, 16(118):3905–3909.
